# Atypical Presentation of Foster Kennedy Syndrome due to Neurocysticercosis: A Rare Case Report

**DOI:** 10.22336/rjo.2024.33

**Published:** 2024

**Authors:** Aparajita Chaudhary, Ruchi Agarwal

**Affiliations:** * Department of Ophthalmology, Moti Lal Nehru Medical College, Prayagraj, Uttar Pradesh, India

**Keywords:** Foster Kennedy syndrome, neurocysticercosis, disc edema, optic atrophy

## Abstract

This case highlights the atypical presentation of Foster-Kennedy syndrome (FKS) associated with Neurocysticercosis (NCC), a prevalent cause of space-occupying lesions in areas endemic to the parasite. We report a newly diagnosed case of NCC in a 13-year-old boy who presented with a one-day history of abnormal movements of the left side of the body and no ocular complaints. Fundus examination of the patient revealed temporal disc pallor and a cup disc ratio (CDR) of 0.6 in the right eye suggesting unilateral optic disc atrophy and a hyperaemic disc with CDR 0.3 and blood vessel tortuosity in the left eye suggesting contralateral impending disc edema, mimicking the classic triad of FKS. He was diagnosed with NCC based on clinical features and radiological findings and was started on Carbamazepine (400 mg), Prednisolone (60 mg), Albendazole (400 mg), Acetazolamide (750 mg), and Vitamin B12 complex.

**Abbreviations:** BCVA = Best Corrected Visual Acuity, CDR = Cup-Disc Ratio, CT = Computed Tomography, FKS = Foster Kennedy Syndrome, IDSA = Infectious Diseases Society of America, ICP = Intracranial Pressure, IOP = Intraocular Pressure, MRI = Magnetic Resonance Imaging, NCC = Neurocysticercosis, OOC = Orbital/Ocular Cysticercosis, OD = Right Eye, OS = Left Eye, OU = Both Eyes, RNFL = Retinal Nerve Fibre Layer, WNL = Within Normal Limits

## Introduction

Foster Kennedy described three symptoms indicative of a space-occupying lesion in the baso-frontal region: ipsilateral optic atrophy, contralateral optic disc edema, and ipsilateral anosmia. Without a space-occupying lesion, these findings may be labeled as Pseudo-Foster Kennedy Syndrome. The aetiology of optic disc pallor is attributed to be secondary to the direct compression of the prechiasmatic optic nerve fibres, and the contralateral disc edema is thought to be caused by increased intracranial pressure (ICP) from the space-occupying mass [**1**].

Foster-Kennedy Syndrome (FKS) has been classically reported due to olfactory groove, falx, sphenoidal wing, or subfrontal meningiomas [**2**]. The other associated intracranial masses are craniopharyngioma, pituitary adenoma, neuroblastoma, nasopharyngeal angiofibroma, aneurysm, and plasmacytoma [**3**].

The most prevalent helminthic infection of the neurological system, neurocysticercosis (NCC), is a leading global cause of acquired epilepsy [**4**]. Seizures are the most common clinical symptom; however, a significant percentage of patients may experience focal deficits, intracranial hypertension, or cognitive decline [**5**].

Orbital/ocular cysticercosis (OOC) is a preventable cause of blindness. Cysticercus cellulosae is the larval stage of T. solium that causes OOC [**6**]. In India, the most common site of the OCC has been reported to be the ocular adnexa [**7**], while in the Western world, it is the posterior segment of the eye. Other ocular sites where the cyst has been found are the subretinal space, vitreous, the subconjunctival space, the anterior segment, and the orbit [**8**].

We present a case of unilateral optic disc atrophy and contralateral impending disc edema attributed to the presence of an ipsilateral cysticercus granuloma. To our knowledge, this is the first case of this form of FKS reported in the literature.

## Case report

A 13-year-old male patient was referred from the Neurology Department to Ophthalmology to rule out ocular cysticercosis. He presented to our department with a one-day history of sudden onset of abnormal movements of the left side of the body. A family member had noticed shaking movements of arms and legs that had lasted for three minutes, followed by lethargy afterward. There was no history of fever, chills, night sweats, weakness, motor or sensory deficits, or vision changes. The history did not reveal any prior trauma, systemic disease, or medication use. On physical examination, the patient was afebrile, conscious, cooperative, and well-oriented to time, place, and person. Cardiovascular, respiratory, and gastrointestinal examinations indicated no apparent abnormalities. On neurological examination, bilateral deep tendon reflexes were +2 (brisk response) and bilateral plantar reflexes were plantar. Cranial nerves and sensory examination results were within normal limits (WNL).

Laboratory examinations revealed a total leucocyte count of 11500 cells/mm3 and an eosinophil count of less than 2 percent. Electroencephalogram and chest X-ray were inconclusive. Computed tomography (CT) demonstrated a ring-like enhancing lesion (size 0.80 x 0.93 cm diameter) with perifocal edema in the right parietal region. These findings were suggestive of a right parietal inflammatory granuloma due to neurocysticercosis. The patient was started on Carbamazepine (400 mg), Prednisolone (60 mg), Albendazole (400 mg), Acetazolamide (750 mg), and Vitamin B12 complex from the Neurology Department.

The patient denied the present ocular complaint. There was no history of blurred vision, transient visual obscuration, diplopia, headache, tinnitus, or anosmia. The best corrected visual acuity (BCVA) was 20/20 in both eyes (OU). Near visual acuity was N6 OU and on examination of color vision on Ishihara plates, 38/38 plates were identified. The pupillary reaction showed normal direct and consensual reflex OU. Intraocular pressure (IOP) was 12.2 mmHg OD and 17.3 mmHg OS. Primary ocular direction revealed orthophoria, extraocular movements were full, and no proptosis was present. Anterior segment examination on slit lamp microscope was WNL OU.

**Fig. 1:**
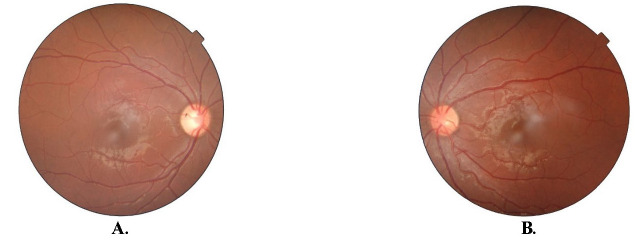
Fundus photo suggestive of optic atrophy OD (**A**) and impending disc edema OS (**B**)

A dilated fundus examination revealed temporal disc pallor and a cup disc ratio (CDR) of 0.6 in the right eye (OD) (**Fig. 1A**). In the left eye (OS), the optic disc was hyperaemic with intact margins with 0.3 CDR and blood vessel tortuosity was present (**Fig. 1B**). Foveal reflex and peripheral retinal appeared normal in each eye. 

**Fig. 2:**
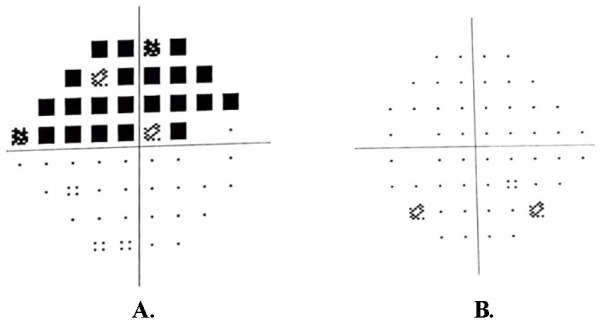
Humphrey 24-2 Visual Field OD (**A**) and OS (**B**)

On Humphrey 24-2 visual field testing, a general sensitivity reduction was present in the superior visual field of the right eye (**Fig. 2A**) and the left eye was WNL (**Fig. 2B**).

**Fig. 3:**
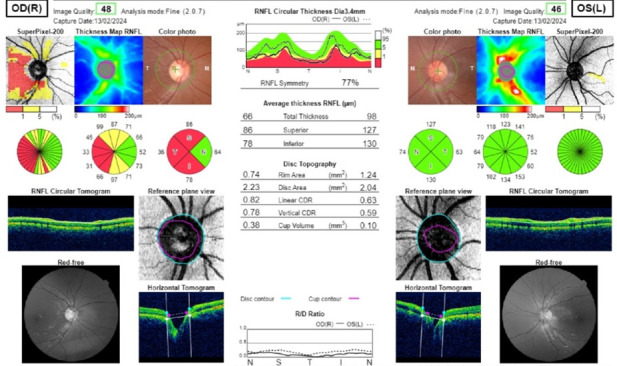
OCT-RNFL image suggesting RNFL thinning superiorly, inferiorly, and temporally in the right eye and fullness of the optic disc with a reduced cup volume in the left eye

Optical coherence tomography (OCT) revealed a total average retinal nerve fibre layer (RNFL) thickness of 66 microns OD and 98 microns OS. Superior, inferior, nasal, and temporal RNFL thickness OD was 86, 78, 64, and 36 microns respectively. It was 127-, 130-, 63- and 74-microns OS. Vertical CDR was 0.78 OD and 0.59 OS and the cup volume was 0.38 OD and 0.10 OS mm3 (**Fig. 3**).

## Discussion

This case reported a patient with contralateral impending disc edema and ipsilateral optic disc pallor who was diagnosed with neurocysticercosis based on clinical and radiological examination. This clinical picture was consistent with an atypical presentation of FKS. In 1911, Robert Foster Kennedy proposed that increased ICP caused contralateral disc edema, whereas ipsilateral optic atrophy was due to direct optic nerve compression [**1**]. In addition to the triad of FKS, other manifestations include headache, emotional lability, cognitive impairment, weakness, nausea, and vomiting [**9**]. In 1962, Tonnis conducted the biggest case series in Germany, wherein 28 of 3,033 individuals with intracranial tumors were discovered to have Foster-Kennedy syndrome. FKS is uncommon and reported to be less than 1% in conjunction with intracranial neoplasms [**10**].

Neurocysticercosis is a prevalent cause of cerebral space-occupying lesions in endemic regions. Cysticercus larvae of Taenia solium cause cysticercosis. It may manifest as spinal involvement, parenchymal, ventricular, or subarachnoid-cisternal [**11**]. Parenchymal cysts might cause seizures, headaches, or localized neurologic impairments whereas, ventricular or subarachnoid-cisternal cysts induce obstructive hydrocephalus due to direct ventricular system blockage by the cyst or secondary to meningeal inflammation. In India, intraventricular and subarachnoid cysts are uncommon [**12**].

In NCC, visual loss is not a common presentation but may occur secondary to optic neuropathy from papilledema. In 1905, three cases of cysticercus-induced postpapilledema optic atrophy were described [**13**]. Five years later, Uhthoff claimed that 1.1% of papilledema patients encountered in his practice were caused by cysticercosis [**14**]. Wilbrand and Saenger [**15**] reported one case of monocular temporal hemianopia and two of homonymous hemianopia. More recently, Sotelo et al. [**16**] reported that 10% of their 763 cysticercus patients had impaired visual acuity and 28% had papilledema. Chang et al. [**17**] analyzed visual loss in 23 patients with cysticercus of whom, 13 had optic neuropathy due largely to papilledema. They described other possible mechanisms of visual loss due to chiasmal involvement from inflammation, compression by large cysts, or retrochiasmal damage by compression or cerebral vasculitis.

Parenchymal cysts on imaging progress from non-enhancing (viable phase) to ring-enhancing (degenerating phase) to calcified nodule (non-viable phase) or complete resolution [**18**]. Parenchymal calcifications are identified best on CT scans while magnetic resonance imaging (MRI) is preferred for temporal and frontal lobe lesions close to the skull base, intraventricular or subarachnoid cyst [**19**]. A drawback of our study is the lack of histopathological or serological evidence. The diagnosis in our patient was made on high clinical suspicion and radiological evidence, as a negative serology result does not rule out the diagnosis of NCC [**19**]. 

The Infectious Diseases Society of America (IDSA) provided the diagnosis and management guidelines for the neurocysticercosis treatment in 2017. The guidelines are based on patient symptoms like headache, seizures, and indication of neurologic involvement [**20**]. In patients with even a single episode of seizure, anticonvulsant drug therapy is recommended as NCC lesions may further act as a nidus for recurrent seizures. Phenytoin, carbamazepine, or levetiracetam can be used. Anti-parasitic therapy with Albendazole or Praziquantel is used for patients with viable and/or degenerating cysts, but should be done under close supervision as initially, the patient may get clinically worse. In case of only calcified lesions, untreated hydrocephalus, or high cyst burden, anti-parasitic therapy is avoided. Degeneration of viable cysts by anti-parasitic therapy can result in seizures, thus adjunctive corticosteroid therapy, most commonly with prednisolone (1 mg/kg/day) or dexamethasone (0.1 mg/kg/day) is recommended [**21**].

Our patient was started on anti-convulsant and anti-parasitic medications, under corticosteroid cover as described in the literature. This patient is being followed up closely so that any progression of his ocular and systemic condition is evaluated.

Cysticercosis is a chronic, self-limited disease, and evaluation with management of ocular manifestations is vital. Although, the treatment is individualized, as elevated ICP can cause optic disc edema, if present it should be treated promptly. Intracranial lesions due to NCC can present as ipsilateral optic atrophy and contralateral disc edema. This combination may be considered a form of Foster-Kennedy syndrome.

## Conclusion

Ophthalmological examination in every patient with neurocysticercosis is essential. Along with adnexal and anterior segment examination, a dilated fundoscopy is vital to look for any optic disc changes because of the neurological lesion. Foster-Kennedy syndrome, although commonly associated with a frontal lobe lesion, can manifest in atypical forms in such patients.

**Conflict of Interest Statement**

The authors state no conflict of interest.

**Informed Consent and Human and Animal Rights Statement**

All appropriate patient consent forms were obtained. In the form, the patient’s attendant has given consent for his child’s images and other clinical information to be reported in the journal. The patient’s attendant understood that his name and initials would not be published and due efforts would be made to conceal the identity, but anonymity cannot be guaranteed.

**Authorization for the use of human subjects**

Ethical approval: The research related to human use complies with all the relevant national regulations, and institutional policies, as per the tenets of the Helsinki Declaration, and has been approved by the ethics committee of Moti Lal Nehru Medical College, Prayagraj, Uttar Pradesh, India (IEC/MLNMC/2024/No.36., Approved on 24/04/2024).

**Acknowledgments**

None.

**Sources of Funding**

None.

**Disclosures**

None.
